# Supramolecular self-assembly and physical-gel formation in disc-like liquid crystals: a scalable predictive model for gelation and an application in photovoltaics

**DOI:** 10.1039/c8ra09278h

**Published:** 2019-02-21

**Authors:** Sehrish Iqbal, Ammar A. Khan

**Affiliations:** Department of Physics, Syed Babar Ali School of Science and Engineering, Lahore University of Management Sciences (LUMS) Sector U, DHA Lahore 54792 Pakistan ammar.ahmed@lums.edu.pk

## Abstract

The application of triphenylene-based discotic liquid crystal derivatives as physical gelators is investigated. In particular, we focus on 2,3,6,7,10,11-hexakis-pentyloxytriphenylene (HAT5) and the longer alkyl chain homologue (HAT6). The driving mechanisms behind and parameter space of non-covalent physical gel formation is studied. A Hansen solubility parameter (HSP) approach is used to predict physical gelation of these disc-like liquid crystalline molecules in a variety of common organic and inorganic solvents important to electrochemical devices. Our results show that HSP analysis is very useful for the prediction of gel formation. The results are transferrable and can form the basis for future investigations into liquid crystalline physical gels. Furthermore, we use acetonitrile as a solvent and apply the gels as electrolytes in dye sensitized solar cells. It is observed that using a binary mixture of gelators results in average photovoltaic power conversion efficiencies as high as 7.21%, compared to a 5.9% reference electrolyte. This is attributed to a reduction in electron recombination at the n-type interface and provides further insight about hybrid gelators. Coupled with an increase in device stability, the results are promising for gel-based dye sensitized solar cells as well as potentially other electrolytic devices such as batteries and supercapacitors.

## Abstract 2

The application of triphenylene-based discotic liquid crystal derivatives as physical gelators is investigated.

## Graphical abstract

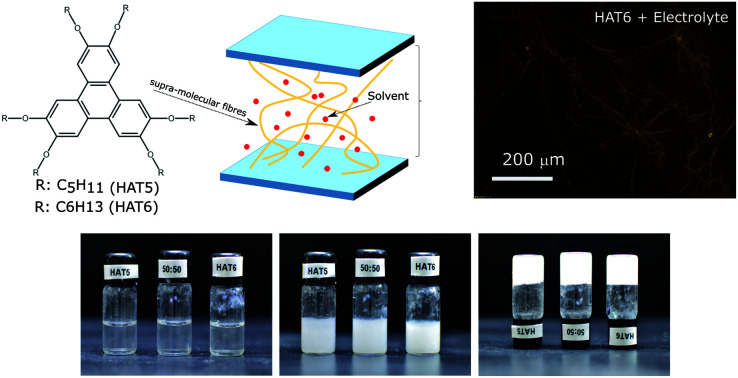

## Introduction

Self-assembled physical gelators have attracted significant attention due to their applications as structural as well as functional materials in optoelectronic devices.^1–3^ The gels consist of a solvent matrix that is restrained within a network of elongated fibres and can be used in optoelectronic devices to give favourable physical properties such as stability, mechanical support, catalysis and self-assembly.^4^ In physical gels the molecular fibres are held together with either hydrogen-bonding, pi-stacking or dispersive inter-molecular forces as opposed to covalent bonds.^2^ A wide variety of small molecules have indeed demonstrated the ability to form physical gels in a diverse range of solvents.^5–7^ It is generally understood that a complex interplay between solvation and precipitation leads to the self-assembly of gelators into long-range interconnected networks (such as elongated fibres) that support and restrict the bulk motion of a liquid solvent.^8,9^ However, a deterministic theoretical predictive model that can accurately predict the gelation of a solvent given with a gelator doesn't, yet, exist and this study attempts to present a modified solubility parameter-based implementation of predicting gel formation with liquid crystalline (LC) gelators.

In a recent study the use of triphenylene discotic liquid crystals (DLCs) as small molecule physical gelators was reported.^10^ It was demonstrated that triphenylene HAT6 (see structure of homologue HAT5 in Fig. 2c.i) forms self-assembled elongated fibres in acetonitrile based electrolytic systems. This system was then utilised as a gel electrolyte in dye sensitized solar cells (DSSCs) and it was shown that the lifetime of DSSCs can be significantly increased due to gel formation, as a direct consequence of a reduction in solvent evaporation due to confinement by the gel phase. Triphenylene HAT6 is a prototypical DLC, and while other recent studies have reported physical gel formation using functionalised DLCs,^11–13^ the ability of use completely unmodified hexaalkyloxy-triphenylene is particularly interesting.^14^ Furthermore, what makes DLCs a particularly interesting class of materials to form gels with is their inherent charge conduction capabilities as molecular wires,^15,16^ self-healing^17^ and low working temperatures. The molecules offer electrically functional as well as mechanical support for the gel-phase depending on how they are engineered and applied.

**Fig. 1:**
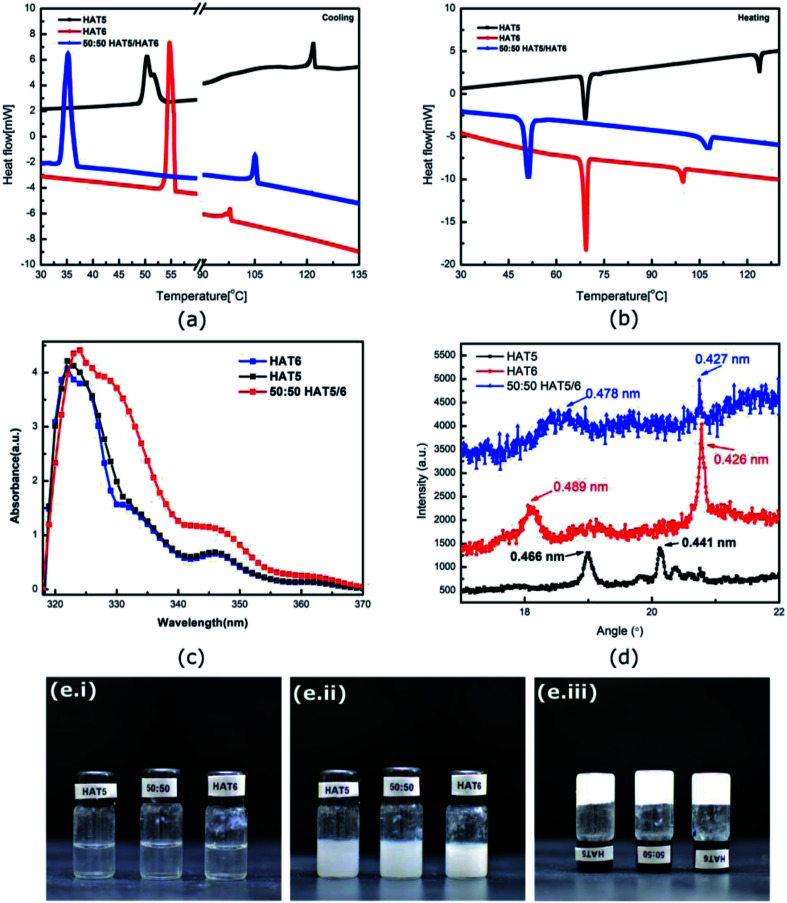
Thermal, UV-Vis and X-ray analysis of the DLCs. DSC curves on (a) cooling and (b) heating of HAT5, HAT6 and a 50 : 50 HAT5/6 mixture. (c) Absorption spectrum of the DLCs dissolved in chlorobenzene and (d) X-ray diffraction of dried drop-casted crystalline films of the DLCs. Photographs of the HAT5, HAT6 and 50 : 50 HAT5/6 gels in acetonitrile in the (e.i) isotropic, (e.ii) gel and (e.iii) inverted state.

**Fig. 2:**
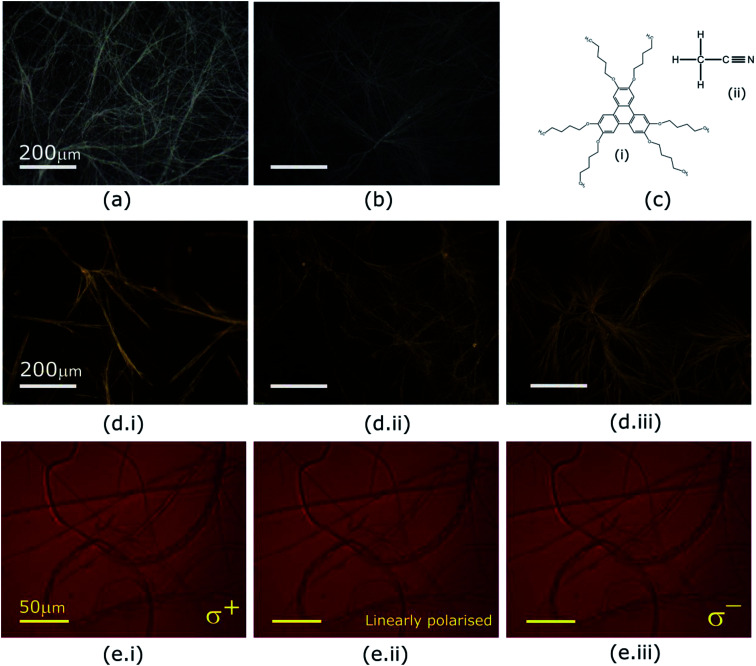
Optical micrographs of dried DLC physical gels observed in dark field microscopy in 60 μm sandwich cells. (a) HAT5 and (b) HAT6 fibers in a utilising acetonitrile as a solvent. (c) Chemical structures of (i) HAT5 and (ii) Acetonitrile. Fibre networks in (d.i) HAT5, (d.ii) HAT6 and (d.iii) 50 : 50 HAT5/6 formed in a the iodide/tri-iodide liquid electrolyte solvent. Optical micrographs of a 25 μm thick sandwich cell filled with a HAT5 physical gel recorded under reflection when illuminated by (e.i) right circular, (e.ii) linear and (e.iii) left circularly polarised light.

In this work we explore three aspects of DLC based physical gel systems to better understand these systems. Firstly, we investigate the effect of alkyl chain length on gel formation. HAT*n* molecules with *n* = 5 (HAT5) and *n* = 6 (HAT6) were tested and compared. We compare thermal stability, gel formation, ionic conductivity and overall device performance when used as electrolytes in dye sensitised solar cells. Secondly, we study gel formation in a variety of different solvents using HAT*n* homologues and report on the driving mechanism of gel formation in light of solvent properties such as polarity and hydrogen bonding sites.

Finally, we perform a detailed and scalable HSP analysis^18^ based on the Nelder–Mead optimization algorithm^19^ and extract the HSP parameters of HAT5 and HAT6 for solubility as well as gelation. Hansen solubility parameters are used to quantify three types of intermolecular forces: dispersive interactions (*δ*_d_), polar interactions (*δ*_p_) and hydrogen bonding (*δ*_h_). Based on the similarity of these three parameters between those of the solvent and those of the gelator, the HSP predicts whether a particular solvent and solute will form a solution.^20^ The HSP approach is an error minimizing method that places solvents and solutes in a three-dimensional (Hansen) space, and is based on the minimization of the Gibbs free energy when a solute and solvent are brought into contact in the bulk. In analogy to their use to predict solubility, recent reports have demonstrated their ability to predict gelation as well.^21^

In this work two independent HSP parameter search schemes based on empirical data are performed. The first is performed to calculate the solvation HSP parameters of the DLC molecules, and the second finds corresponding pseudo-HSP parameters for gelation.^5^ This allows us to make independent solubility and gelation spheres using our empirical data. More interestingly however, we demonstrate that the spheres can then be used to predict gelation in a wide range of untested solvents, being the first study of its kind for functional discotic liquid crystals. The application of the HSP approach to mixtures of solvents is also presented, and we find that the HSP parameters are consistent with experiment in predicting gelation of solvents. Our results can be used to predict if prototypical triphenylene DLCs will gelate a given solvent in an electrochemical device, but more importantly, the approach can be applied to a vast library of untested calamitic as well as discotic^22^ liquid crystalline molecules, each offering selective material and physical advantages.

## Materials and cell fabrication

2,3,6,7,10,11-Hexakis-hexyloxytriphenylene (HAT6) and 2,3,6,7,10,11-hexakis-pentyloxytriphenylene (HAT5) (SYNTHON Chemicals) DLC were used as received. A 50 : 50 wt% HAT5/6 mixture was prepared by dissolving equal amounts in chlorobenzene and evaporating the solvent to leave a homogeneously mixed mixture. Acetonitrile (Sigma Aldrich) was used as a solvent for the redox electrolyte used in DSSCs. The iodide/tri-iodide redox liquid electrolyte was prepared by mixing 0.5 M 4-*tert*-butylpyridine (Sigma Aldrich), 0.5 M lithium iodide (LiI) (Sigma Aldrich), 0.05 M iodine (I_2_) (Sigma Aldrich) in acetonitrile. The contents were stirred until homogeneous mixing.

Fluorine-doped tin oxide FTO glass (GreatCell Solar) was used as a substrate for both the anode and cathode for DSSCs. For DSSC photoanodes, a compact (blocking) layer of TiO_2_ was prepared by spin-coating a titanium iso-propoxide solution (25 μL, 2 M HCl (Fisher) and 250 μL titanium butoxide in 6 ml anhydrous isopropyl alcohol (Sigma Aldrich)). To prepare mesoporous titanium dioxide (TiO_2_) (Dyesol 18 NR-T) was used. The anode was masked using a circular 0.282 cm^2^ aperture to control the device active area. Platinum paste (PT-1 Dyesol) was used a catalyst for electrolyte regeneration. The paste was spin-coated on an FTO substrate at 3000 rpm and then sintered at 500 °C in a furnace.

To prepare test cells for electronic conductivity measurements, an FTO–Pt/Ti electrode configuration was utilised. One of the electrodes was patterned *via* photolithography using an AZ1512 positive photoresist, and a 50 nm platinum (Pt) on 5 nm titanium (Ti) layer was sputtered on the developed surface followed by lift-off using acetone. The second electrode was FTO coated glass, and the two substrates were separated using 25 μm Surlyn film to obtain uniform thickness across the devices. The test cells were filled at room temperature with a heated-isotropic electrolyte, such that *in situ* gel formation occurred in the test cells.

## Experimental and simulation methods

Optical microscopy was used to visualise gel textures of HAT5, HAT6 and 50 : 50 HAT5/6 in capillary filled 60 μm sandwich cells. For dark-field observations an optical microscope (Olympus BX-51) was utilised in reflection mode. For measurements of circular dichroism, a custom microscope setup with a 20× objective was utilised. A while light source (Thorlabs QTH10) was filtered using a 650 nm long pass filter, followed by a linear polarizer and 670 nm *λ*/4 plate to generate left and right circular light for illumination. A UV-Vis spectrometer (Shimadzu UV-1800) was used to record absorption spectra of the pure LC mixtures in solution. Powder X-ray diffraction (XRD) measurements were performed using a Bruker (Bruker D2 Phaser) *λ* = 1.54 Å XRD. For XRD sample preparation a heated isotropic gel sample of each of the samples was drop-casted onto a glass slide and allowed to dry in ambient conditions, leaving behind an interconnected DLC crystalline fibre network/mesh that was then probed using X-rays. To measure phase transition temperatures, a Differential Scanning Calorimeter (DSC) was utilised (Perkin Elmer DSC 8000), at heating and cooling scan rates of 5 °C min^−1^.

A Keithley 2400-series source meter is used to perform current–voltage (*IV*) sweep-measurements in patterned electrode out-of-plane devices to measure ionic conductivity in a sandwich cell configuration. Open circuit voltage decay (OCVD) measurements to estimate electron lifetime in the n-type semiconducting TiO_2_ were performed using white light to simulate open circuit conditions and a digital oscilloscope (Agilent) was used to record the voltage decay as the light was switched off. To perform Electrochemical Impedance Spectroscopy an Autolab impedance analyser was used to measure impedance as a function of frequency. In both OCVD as well as 25 μm thick, un-encapsulated DSSCs were tested. Photovoltaic power conversion efficiency of gel-based DSSCs are measured using an AM1.5 calibrated solar simulator (Photo Emission Tech, CT50AAA). The solar simulator involved real-time monitoring of the light intensity to provide accurate measurements. For HSP analysis, to determine HSP and pseudo-HSP parameters for solvation and gelation, a Matlab script^19^ was used without modification (the HSP data for the solvents utilised in this study were substituted).

## Experimental results and discussion

HAT5 and HAT6 form hexagonal columnar mesophases (liquid crystalline phase) when heated from room temperature (or cooled from isotropic). In the mesophasic temperature region, the molecules align into columns with the columnar directors pointing normal to the conjugated cores of the molecules. It has been shown that triphenylene DLCs can be used in organic semiconducting applications owing to high charge carrier mobility supported by the columnar morphology. Optical absorption data (Fig. 1c) confirms that the materials indeed have high optical bandgaps >3.5 eV, so room temperature charge densities are indeed low. However, it is the ordered morphology and high charge carrier mobility that makes DLCs promising for device applications.

The phase transition temperatures of the materials were measured using DSC (Fig. 1a and b). The crystalline (K) to columnar liquid crystal (LC) temperature for HAT5 and HAT6 was measured to be similar, however, the LC to isotropic temperature was recorded to be 116 °C in the case of HAT5 and 102 °C in the case of HAT6. The higher clearing temperature is a result of the shorter alkyl chain in the case of HAT5.^23^ Flexible alkyl chains add fluidity and lower the clearing temperature. However, while the shorter alkyl chain leads to higher working temperatures, the alkyl chains themselves are electrically insulting, it has been shown in previous studies that shorter-chain triphenylene derivatives support higher charge carrier mobilities due to greater π–π overlap of the polyaromatic cores.^24^ From both Fig. 1a and b (illustrating cooling and heating DSC curves, respectively), it is observed that the 50 : 50 mixture has well-defined phase transitions, indicating uniform mixing of the two DLCs. In a previous study,^10^ we observed that HAT6 and the longer chain homologue HAT10, when mixed together and deposited in films actually form separate domains. The results in the previous study were tested using grazing incidence wide-angle X-ray spectroscopy.^25^ At present, it is not obvious whether HAT5 and HAT6 form intercalated hybrid crystals or whether they form separate crystalline domains. From the DSC data, homogeneous mixing is confirmed. Interestingly, supercooling is apparent in Fig. 1a in the 50 : 50 mixture (where the LC–K transition is delayed), whereas on heating the clearing temperature is intermediate of HAT5 and HAT6.

Physical gels were prepared by adding 5 mg of HAT5, HAT6 or 50 : 50 HAT5/6 to 1 ml of solvent/electrolyte. The mixture was heated to ≈60 °C in an oven and then cooled. A fibre network was formed on cooling (in solvents that support gelation) and the solution turned from clear to a turbid gel. The gelation process is illustrated in the photographs in Fig. 1e. The molarity of the gelators was 6.71 mM, 6.03 mM and 6.37 mM for HAT5, HAT6 and 50 : 50 HAT5/6, respectively, and is above the critical gelator concentration. On heating, the gel phase was found to be stable up to a temperature of 43 ± 0.5 °C (HAT5 in acetonitrile), 55 °C ± 0.5 (HAT6 in acetonitrile) and 46 ± 0.5 °C (50 : 50 HAT5/6 in acetonitrile) where the gel begins to break (bi-phasic region), and further heating up to 46 ± 0.5 °C, 59 ± 0.5 °C and 53 ± 0.5 °C, respectively, resulted in a complete isotropic phase that was optically clear (similar to Fig. 1e.i).

Optical textures of HAT6, HAT5 and a 50 : 50 HAT6/HAT5 mixture physical gels in an acetonitrile solvent are shown in Fig. 2, under dark-field observation. An interconnected network of thin fibres was observed in all three devices that was stable even after removal of the solvent/electrolyte.^14^ The gels can be repeatedly cycled by heating to the isotropic phase followed by cooling and nucleation of the fibres to form the gel.^26^ The morphology, structure and dimensions of the fibres have been found to depend upon cooling rates, thickness of the cavity (when the gel is cooled in a sandwich cell structure), as well as the surface chemistry. In addition, the solvent also plays a major role in determining the gel morphology. Certain solvents are completely averse to gelation, and either solvation or precipitation result. The solvents that do form a gel determine the overall stability of the physical-gel and the DLC network through a complex set of interactions^12^ that include repulsive as well as attractive electrostatic forces that minimise the free energy in a way such that gel formation is preferred. Furthermore, XRD scans were performed on drop-casted (and subsequently dried) physical gels of HAT5, HAT6 and the binary mixture in acetonitrile. There is some crystallization on drying and the original fibre morphology is modified, and an intermediate fibre-LC texture is scanned. Fig. 1d shows peaks corresponding to intra-columnar spacing and are mostly consistent with previous analysis.^12,25^

The optical properties of the supramolecular fibres were further investigated under illumination by circularly polarised light. The triphenylene HAT5/6 derivatives are optically birefringent and it is likely that there is some twisting of the molecular directors along the axis of the fibres. In order to study this effect a HAT5 physical gel in acetonitrile was examined under right/left circular and linearly polarised light, and the results are summarised in Fig. 2e. Interestingly, no dichroism is apparent at the wavelengths tested (>650 nm). It is hypothesized that this is due to a difference in length scales, where the director has a period on the order of microns, two–three orders of magnitude greater than the wavelength of visible light. High resolution small-angle X-ray scattering experiments are recommended in future studies to further probe the microstructure and understand the self-assembly of the fibres.

On comparing optical micrographs of the fibre network in HAT5 and the longer alkyl chain DLC HAT6 (Fig. 2) it was repeatedly found that the fibre network is in HAT5 is composed of thicker fibre aggregates as compared to HAT6, where the fibre network was composed of relatively finer ‘threads’. Furthermore, we found that HAT10, where the alkyl chains are C_10_H_21_ precipitates out in acetonitrile as well as ethanol. So, there is a strong dependence of the alkyl chain length on the ability to form a physical gel. Researchers looking to engineer gel phases for photovoltaic and battery applications must consider the effect of alkyl chain length, as it is only the correct interplay of attractive and repulsive forces, polar and non-polar species that will yield a stable gel phase.

**Fig. 3:**
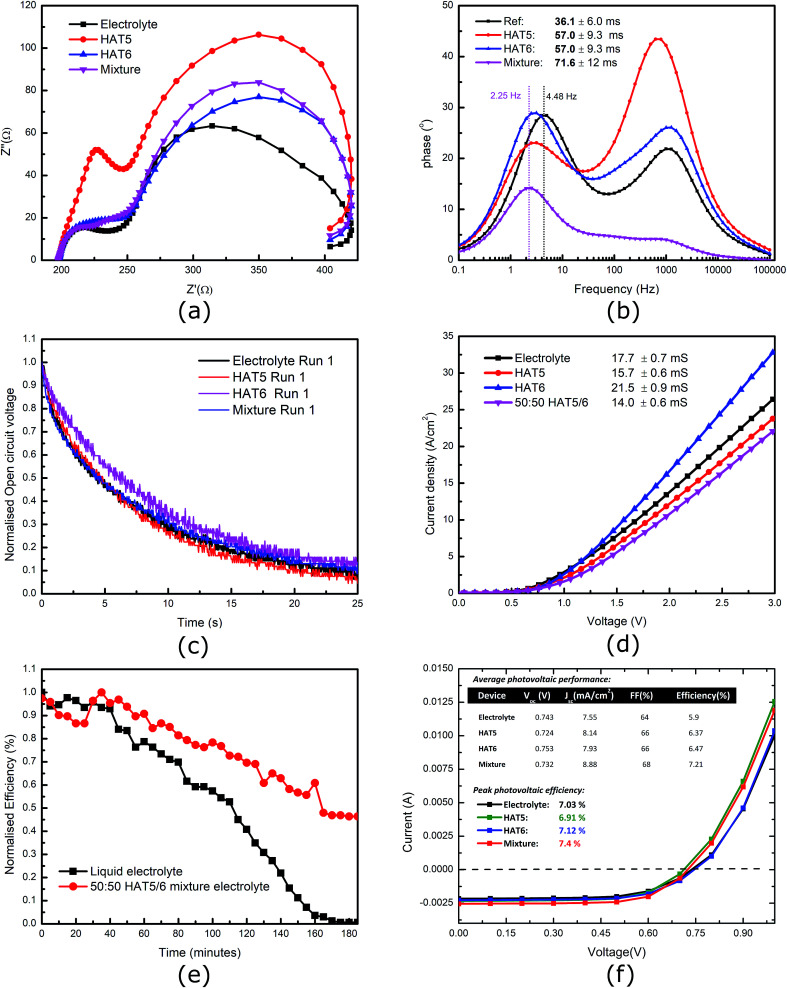
Impedance spectroscopy, electronic conductivity and photovoltaic analysis. (a) Nyquist plot of impedance spectra of DSSCs on sweeping frequencies from 0.1 Hz to 100 kHz. (b) Corresponding phase as a function of applied small signal frequency. (c) OCVD measurements of DSSCs filled with different electrolytes. (d) Electronic conductivity of the gel electrolytes (I^−^/I_3_^−^ redox system) measured in 25 μm thick patterned sandwich cell devices (Pt–FTO). (e) Evolution of device efficiency with time on un-encapsulated liquid electrolyte and 50 : 50 HAT5/6 gel electrolyte DSSCs. (f) A summary of photovoltaic results of DSSCs filled with the gel electrolytes with a liquid electrolyte as a reference. (Inset) Average solar cell parameters across five devices of each type.

The HAT5, HAT6 and HAT5/6 driven physical gels form in pure as well as electrolytic acetonitrile, so it is interesting to see how they compare in a device when used as a key component such a charge transfer electrolyte. Subsequently, DSSCs were fabricated using the three gel electrolytes and keeping a liquid electrolyte as a reference. The cells were tested in a calibrated solar simulator and the results are summarized in Fig. 3f. Five devices of each type were tested, and results for average photovoltaic efficiencies as well as peak PCE of the best devices are summarized (inset Fig. 3f). Devices filled with gel electrolytes were measured to be, on average, more efficient that the reference liquid electrolyte. A PCE of 5.9% was measured using the reference electrolyte, whereas the average PCE of devices filled with the 50 : 50 HAT5/6 mixture was 7.21%. This is a significant improvement indeed, in this controlled trial. This is particularly interesting as the binary mixture forms self-assembled fibres just like pure HAT5 and HAT6 physical gels, and the fibres are either composed of mixed HAT5/6 fibres or separate fibres of HAT5 and HAT6 each contributing their own selective benefits. Further studies are required to probe the molecular composition as well as packing of the micro-fibres in more detail. Physical gels have been shown to improve lifetime,^10,27^ and present a solution to the long-standing electrolyte evaporation issue of DSSCs utilising acetonitrile as a solvent.

Furthermore, electrical conductivity measurements of the iodide/tri-iodide (I^−^/I_3_^−^) liquid electrolyte (reference and gels) were performed and the results are summarised in (Fig. 3d). It can be seen that there is no loss of conductivity on gel formation. This is because the solvent as well as ionic species are free to move in the solvent matrix, and the gelator fibres are phase-separated. In addition, preliminary device lifetime measurements were performed on un-encapsulated DSSCs. In previous studies, it was demonstrated that a HAT6 gel electrolyte leads to significant increases in device stability.^10^ Here, we compare a 50 : 50 HAT5/6 gel electrolyte with a reference liquid electrolyte, and the results are demonstrated in Fig. 3e It is observed that device lifetime is definitely longer in the gel electrolyte, as expected. Future studies that compare long term stability of encapsulated solar cells operating under maximum power point conditions are required to give more quantitative results (and to account for statistical variations across devices) as well as determine relative improvements between the different gel electrolytes. In the area of DSSCs, stability and efficiency are key, and more studies are required to make more substantial progress.

To further investigate the dynamics of charge transfer processes taking place at interfaces in the DSSCs, electrochemical impedance spectroscopy was performed on the DSSCs in open circuit conditions (0.7 V bias) in the dark. Similar to previous studies^28,29^ the EIS results, particularly the phase plots (Fig. 3b) can be used to estimate the electron lifetime in the mesoporous TiO_2_ using the relation 
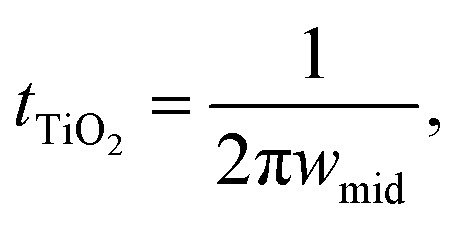
 where *w*_mid_ is the frequency of the middle phase peak, representing processes taking place in the TiO_2_. EIS spectra is illustrated in Fig. 3a and corresponding phase angle is plotted in Fig. 3b. There is a left shift in the peak position in the gel phases when compared to the reference electrolyte. Furthermore, the degree of left shift is directly correlated with the photovoltaic efficiencies reported in the table of average device efficiencies (inset Fig. 3f). The charge carrier lifetime is calculated to be 36.1 ± 6 ms in the reference electrolyte and 71.6 ± 12 ms in DSSCs filled with the hybrid mixture. Higher lifetimes mean slower recombination, and correspondingly higher short-circuit currents, as seen in the summarized photovoltaic results. In a previous study we showed that HAT6 fibres shield the surface charge on the TiO_2_, reducing recombination with the electrolyte. In this work we find that the effect is much more pronounced in the HAT5–HAT6 mixture, open circuit voltage decay measurements were performed to further study electron lifetime in the TiO_2_, and the results are summarized in Fig. 3c. The OCVD results show a relatively smaller improvement in the electron lifetime as compared to the EIS results, however both results point towards a net increase in electron lifetime in the DSSCs on gel formation.

## Hansen solubility analysis

**Fig. 4:**
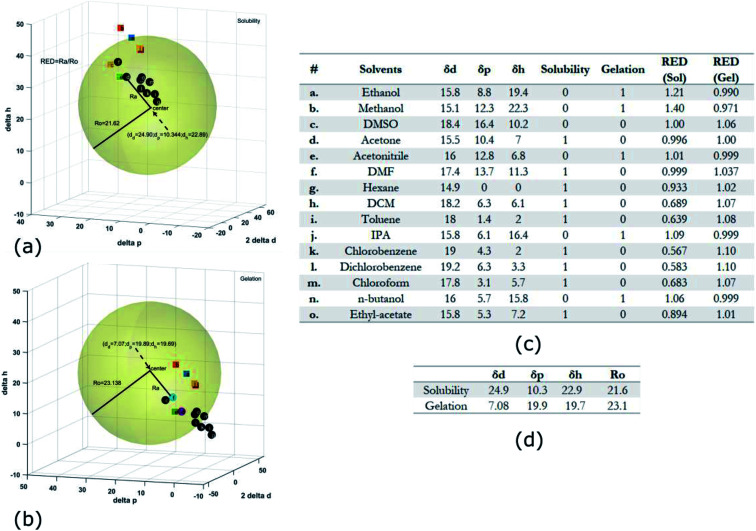
HSP analysis and determination of solubility and gelation parameters (measured in
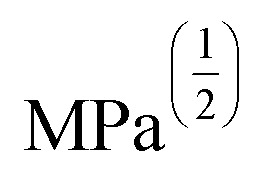
) of HAT6 using empirical data. (a) Solubility sphere with an interaction radius of 
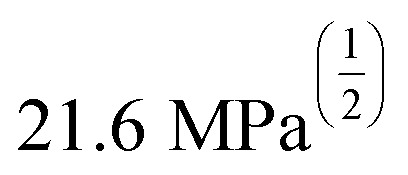
 centred on the solubility parameters of HAT6. (b) Gelation sphere with an interaction radius of 
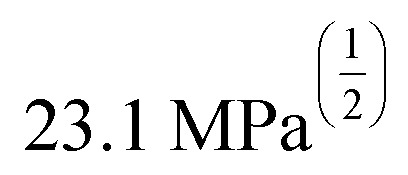
 centred on the gelation parameters of HAT6. (c) A summary of HSP parameters of a set of fifteen solvents, (data taken from ref. 19) ‘0’ corresponding to no gel/solution and ‘1’ corresponds to the formation of a gel or solution in the respective column. (d) HSP parameters of HAT6 calculated using the Matlab script^19^).

Gel formation in a typical DLC such as triphenylene HAT5 and HAT6 can be understood by considering the inter-solvent and intra-columnar interactions of three motifs on the molecules. These motifs can are identified as the conjugated poly-aromatic central core, the linking ether functional group and the flexible alkyl chain (see Fig. 2c.i). The molecules are non-polar and precipitation is observed in a highly polar solvent such as water.^23^ Similarly, the molecules completely dissolve in non-polar solvents such as benzene, and a full list of tested solvents and corresponding results in shown in Fig. 4c.

While a classification into polar and non-polar solvents would be convenient, experimental data on gelation requires careful analysis. Alcohols such as isopropanol, ethanol, methanol and *n*-butanol supported gel formation, and it is hypothesized this the primary mechanism behind stabilization is hydrogen bonding between the hydroxyl group in the alcohols and the electronegative oxygen site within the ether linking group in the HAT5/6 molecules.^13,30^

In acetonitrile the polar nitrile group (Fig. 2c.ii) presents a site for attraction between the highly electronegative nitrogen and the partially positive carbon-linking groups next to the ether-link oxygen (Fig. 2c.i). Furthermore, the electropositive carbon in the nitrile bond can interact with the oxygen in the HAT5/6 molecules to aid stabilization. The attractive forces are counterbalanced by the repulsive polar-on-polar interactions such that the net result favours neither solvation nor precipitation, and support an interconnected network of elongated LC fibres.

**Fig. 5:**
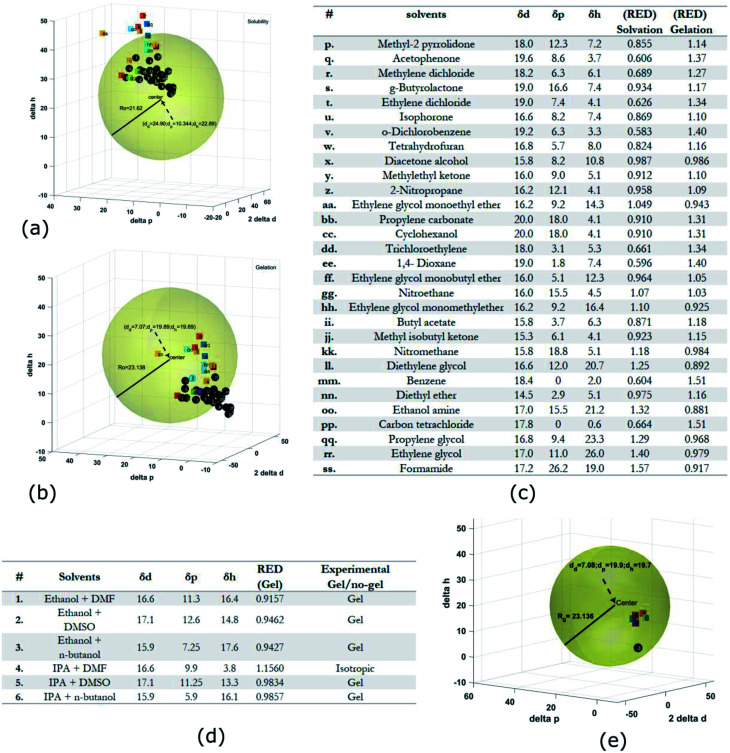
Predicted solubility and gelation spheres of HAT6 using the calculated HSP parameters (measured in
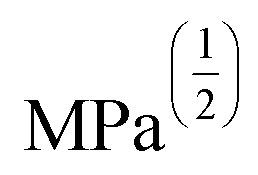
). (a) Solubility sphere centred on HAT6 and (b) gelation sphere. Solvents inside are predicted to dissolve and form a gel, respectively. (c) A summary of HSP parameters of the plotted solvents and the calculated values of the relative energy difference for solvation and gelation. (d) A summary of HSP parameters as well as composition of 50 : 50 vol% binary mixtures. (e) Gelation sphere indicating the gelation (points inside the sphere) of binary mixtures. All points with the exception of the circle numbered ‘4’ lie within the sphere.

The HSP approach towards solvation/gelation categorizes the physical interactions of a solute and solvent into three parameters. These are the dispersion parameter (*δ*_d_), the polar parameter (*δ*_p_) and the hydrogen bonding parameter (*δ*_h_). Unknown materials are classified according to their solubility characteristics in known solvents, and reference tables can be readily accessed.^31^ Reference data for some solvents of interest is summarized Fig. 4c and 5c.^19^ The three coordinates are used to form a three-dimensional solubility space with (2*δ*_d_, *δ*_p_, *δ*_h_) as the orthogonal axes.

We experimentally determined the solubility parameters for HAT5 and HAT6 by adding 5 mg of HAT5/6 to 1 ml of different solvents and noting the results in terms of solvation/gelation/precipitation. The results are summarized in Fig. 4c. Subsequently, a Matlab-based HSP determination script^19^ was used to find the HSP parameters of the DLCs the best fits the experimental results. The script was used without modification by inserting the HSP parameters of the solvents that were empirically tested (Fig. 4c). The optimization function outputs the HSP parameters, as well as an interaction radius *R*_o_ for the given distribution of solvents in the solubility space. The interaction radius *R*_o_ is the radius of the ‘solubility sphere’, shown in Fig. 4a The HSP parameters of HAT5 and HAT6 are taken as the centre of the sphere and the distance of a point (a solvent) from the centre of the sphere is calculated as: *R*_a_^2^ = 4(*δ*_d_ − *δ*^s^_d_)^2^ + (*δ*_p_ − *δ*^s^_p_)^2^ + (*δ*_h_ − *δ*^s^_h_)^2^. The ratio 
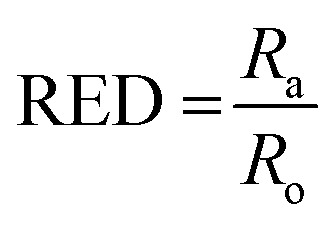
 (relative energy difference) determines whether a solvent lies inside or outside the solubility sphere (see Fig. 4a).

In an analogous approach, we extended the HSP solubility approach to gelation, similar to previous studies,^5^ where organogel formation was predicted in pure as well as solvent mixtures.^32^ The same software script that was previously utilised for the computation of (2*δ*^s^_d_, *δ*^s^_p_*δ*^s^_h_) for solvation, is now used for calculating ‘pseudo’ HSP parameters for gelation (2*δ*^g^_d_, *δ*^g^_p_*δ*^g^_h_). The key difference is that the yes ‘1’ and no ‘0’ data is encoded as the empirical observation of gelation as opposed to solvation. As a result, we obtain three parameters for HAT5/6 that specify the location of the gelation sphere in the gelation space. Once again, an interaction radius *R*_o_ specifies the radius of the gelation sphere that encloses those points (solvents) that support gelation. The results are summarized in Fig. 4b.

Subsequently, once the solubility and gelation spheres are defined, it is possible to map HSP data of unknown solvents into the solvation and gelation spaces. The table in Fig. 5c summarizes our results for a list of commonly used solvents. The HSP solubility parameters have been quoted from ref. 19. The relative energy difference has been computed by calculating the distance of each solvent from the center of the solvation and gelation sphere of HAT5/6. RED values less than one in the case of solvation predict that a particular solvent will dissolve HAT5/6 and RED value greater than one predicts that gelation/precipitation will occur.

It can be seen (Fig. 5a) that several untested solvents (see Fig. 5c) can potentially be used to form physical gels with triphenylene DLCs HAT5/6. It is interesting that almost all of the solvents that lie within the gelation sphere have an OH-group, such as di-acetone alcohol and ethylene glycol, so we hypothesize that hydrogen bonding is a significant stabilizing factor, at least in the case of ether-linked triphenylene LCs. It would be very interesting to study gel formation of larger core DLCs such as coronenes and pyrenes, as the large cores will support inherently higher charge carrier mobility as well as un-doped carrier density.

The HSP analysis can also be extended to mixtures of solvents.^33^ The analysis was performed for three mixtures of ethanol and IPA, and the results are summarized in the table in Fig. 5d. Three mixtures were made each with DMF, DMSO, and *n*-butanol, as 50 : 50 volume fractions. The HSP parameters of the mixtures were estimated from the HSP parameters of the pure solvents using the method described in ref. 31 and 33. The reason for choosing these particular solvents was that in the initial analysis presented in Fig. 4c DMF lead to the formation of a clear isotropic liquid, DMSO lead to the formation of a precipitate and *n*-butanol supported a physical gel. Interestingly, on mixture formation it is observed that a gel is formed in all binary mixtures, with the sole exception of IPA + DMF. The simulation results in Fig. 5e agree fully with experiment, further strengthening the predictive power of HSP as a reliable metric for determining supramolecular DLC gel formation.

This approach is beneficial as device preparation (such as batteries, solar cells and super-capacitors) involves looking for solvents with relevant physical properties. If a physical gel phase is desired then the approach described here can be used to quickly narrow the search for the solvent that will best suit the engineering needs of a particular device, with minimal hit and trial. The vast libraries of HSP parameters can serve as a valuable resource and all researchers have to do is to determine the HSP parameters of a gelator/liquid crystalline material and then proceed towards device testing and development.

## Conclusions

It is demonstrated that triphenylene DLC HAT5, HAT6 and a 50 : 50 binary mixture can be used to form physical gels in a variety of solvents. In addition to alkyl chain length of the DLCs, the hydrogen bonding ability of the solvent is identified as one of the main stabilising interaction in HAT*n* liquid crystalline physical gels. On testing for photovoltaic performance in DSSCs, the binary mixture shows a remarkable improvement in power conversion efficiency when used as a redox electrolyte. The microstructure/morphology of the mixed-gelator gels favours device efficiency, and this is very interesting from a device application perspective. Careful X-ray, high resolution atomic force microscopy and transmission electron microscopy characterizations are recommended in future studies. The photovoltaic performance correlated well with electron lifetime measurements in the TiO_2_, suggesting that the hybrid gel helped minimize electron recombination through dielectric screening.

HAT5, HAT6 and their binary mixture exhibited nearly identical macroscopic gelation and solubility properties, with differences only in the microscopic geometries of the elongated fibres. HSP analysis resulted in identical HSP parameters for solvation and gelation for the two homologues. Thermal stability (gel-isotropic transition temperature) was found to be higher in HAT6 as compared to HAT5, but the much longer chain homologue HAT10 however was found to precipitate in acetonitrile and ethanol, so alkyl chain length is very important when it comes to gelation, in addition to hydrogen-bonding groups between the solvent and DLCs. Once HSP spheres were established, the results were then used as a predictive tool to determine gelation, solvation or precipitation in unknown solvents. This approach can be extended to a wide variety of poly-aromatic organic semiconductors, particularly other DLC systems of importance in optoelectronic devices such as phthalocyanines and coronenes. In addition to DSSCs, batteries, super capacitors, fuel-cells and electrochemical sensors can benefit from reliable physical gelation in appropriate solvents. Future studies incorporating larger core DLCs can potentially harness the inherent conductivity properties of the DLCs with benefits of physical-gels to fabricate a new class of functional soft-matter optoelectronic devices.

## Conflicts of interest

There are no conflicts to declare.

## Supplementary Material
